# Crosstalk Between Circulating Follicular T Helper Cells and Regulatory B Cells in Children With Extrinsic Atopic Dermatitis

**DOI:** 10.3389/fimmu.2021.785549

**Published:** 2021-11-30

**Authors:** Jinqiu Jiang, Shi Yan, Xiaoying Zhou, Jinghua Zhou, Xiaoming Bai, Qi Tan, Yunqiu Xia, Hua Wang, Xiaoyan Luo

**Affiliations:** ^1^ Department of Dermatology, Children’s Hospital of Chongqing Medical University, Chongqing, China; ^2^ Chongqing Key Laboratory of Child Infection and Immunity, Children’s Hospital of Chongqing Medical University, Chongqing, China; ^3^ Ministry of Education Key Laboratory of Child Development and Disorders, Children’s Hospital of Chongqing Medical University, Chongqing, China; ^4^ National Clinical Research Center for Child Health and Disorders, Children’s Hospital of Chongqing Medical University, Chongqing, China

**Keywords:** atopic dermatitis, regulatory B cells, T follicular helper cells, IL-10, immunoglobulin E

## Abstract

Atopic dermatitis (AD) in early childhood is often the initial manifestation of allergic disease associated with high IgE. Accumulating evidences show that follicular helper T (Tfh) cells play a critical role in promoting B cell differentiation and IgE production, human regulatory B (Breg) cells participate in immunomodulatory processes and inhibition of allergic inflammation. However, the roles and interactions between IL-10-producing Breg cells and Tfh cells in childhood AD are unclear. In this study, we found that the percentage of CD19^+^IL-10^+^ Breg cells in children with extrinsic AD was significantly lower than that in age-matched healthy controls, and that it correlated negatively with enhanced CD4^+^CXCR5^+^PD-1^+^ICOS^+^ circulating Tfh cell responses and increased disease activity; however, there was no significant correlation with serum total IgE levels. A co-culture system revealed that Breg cells from patients with extrinsic AD cannot effectively inhibit differentiation of Tfh cells in an IL-10 dependent manner. Abnormal pSTAT3 signaling induced *via* Toll-like receptors (TLR), but not the B-cell receptor (BCR) signaling, might contribute to the defect of Breg cells in AD. Taken together, these observations demonstrate an important role for IL-10-producing Breg cells in inhibiting Tfh cell differentiation, and suggest that they may participate in the pathogenesis of AD.

## Introduction

Atopic dermatitis (AD) is the most common chronic inflammatory skin disease, affecting up to 20% of children and 1–3% of adults worldwide (1, 2). It is characterized by severe itching, eczema-like lesions, and hypersensitivity to environmental substances. Despite its high incidence and negative impact on patients and their families, the pathogenesis of AD still remains not fully understood (3). AD has been categorized into extrinsic (EAD) and intrinsic (IAD) clinical phenotypes based on serum total IgE levels and the presence/absence of specific IgE to environmental and food allergens. EAD and IAD represent approximately 80% and 20% of AD patients, respectively (4). IAD has a similar clinical phenotype to EAD, but is characterized by female predominance, normal total serum IgE levels, absence of atopic diseases, and normal skin barrier function (5). Immunologically, IAD is associated with higher activation of Th17 and Th22 cells than EAD (6). EAD has a Th2-polarized immunophenotype with high levels of total IgE and allergen-specific IgE, and is associated with an increased risk of other atopic diseases, including asthma, allergic rhinitis (AR), and food allergies (4, 7). Many studies reporting the immunological characteristics of EAD have demonstrated that the high IgE level in EAD patients is not due solely to Th2 bias (3, 8); however, the underlying mechanisms still remain unclear. Children with AD are in the early stages of the “atopic march”; therefore, research on the regulation of IgE production of different types of AD will help us to better understand the development of subsequent allergic diseases.

T follicular helper cells (Tfh cells) are a subset of CD4^+^T lymphocytes that migrate to B cell follicles and promote the cognate B–T cell interaction; they then provide germinal center B cells with survival and differentiation signals that are essential for production of high affinity antibodies (9). Tfh cells are involved in the development of autoimmune and allergic diseases, including rheumatoid arthritis, systemic lupus erythematosus, and allergic asthma (10). Mouse models of allergic asthma and food allergy show that CXCR5^+^Tfh cells rather than CXCR5^-^Th2 cells are needed to support IgE production (11, 12). Up-regulation of Tfh cell activity occurs in patients with allergic diseases, including a phenotype that skews toward type 2 Tfh (Tfh2) cells and IL-13-producing Tfh (Tfh13) cells (12, 13). In children with AD, CXCR5^+^ICOS^+^PD-1^+^ circulating Tfh (cTfh) cells expand and increase production of IL-21, which correlates positively with disease severity (14). Although evidence shows that costimulatory ligands expressed by B cells play a role in promoting Tfh cell differentiation (15), it is unclear whether B cells can also regulate Tfh cell differentiation in AD patients.

Regulatory B cells (Bregs) negatively regulate immune responses *via* release of IL-10, TGF-β, and IL-35. They play an important role in a variety of autoimmune and inflammatory diseases by inhibiting activation of CD4^+^ T cells (16, 17). Human Breg cells are identified mainly by expression of IL-10, which is enriched in CD24^hi^CD38^hi^ transitional B cells (18) and CD24^hi^CD27^+^ memory B cells (19). A recent study showed that human Breg cells control Tfh cell maturation and inhibit Tfh cell-mediated antibody secretion (20). Deficient Breg cells cannot effectively inhibit the maturation and function of Tfh cells, which leads to autoimmune diseases (21). During development of primary Sjögren’s syndrome (pSS), Breg cells are critical for restraining the Tfh cell responses in an IL-10 dependent manner (21).

The interaction between IL-10^+^ Breg cells and Tfh cells has not been investigated in AD. Here, we show that the percentage of IL-10^+^ Breg cells in children with extrinsic AD is significantly reduced, and correlates negatively with enhanced Tfh cell responses and increased disease activity. A co-culture system revealed that Breg cells from patients with extrinsic AD cannot effectively inhibit differentiation of Tfh cells in an IL-10 dependent manner.

## Materials And Methods

### Patients and Control Subjects

A total of 32 patients with AD fulfilled the diagnostic criteria of Hanifin and Rajka (22) and 32 healthy age-matched controls (HC) without a family history of asthma or other atopic disease were enrolled in the study. Participants who had received systemic corticosteroids or immunosuppressant and had ongoing infections within one month before the study were excluded. SCORAD (scoring atopic dermatitis) index was used to evaluate AD disease severity which sub-divided the patients into mild (scores<25), moderate (25–50) and severe (>50). Based on total serum IgE levels, AD was classified as intrinsic (IgE ≤ 150 IU/mL) or extrinsic (IgE > 150 IU/mL). Demographic data are listed in **Table 1**. The study was approved by the local institutional review boards, and all participants (or their legal guardians) provided informed consent.

**Table 1:** Clinical features and demographics of healthy controls and patients with AD.

	Healthy Controls	AD	
		Extrinsic AD	Intrinsic AD
Total number of subjects	32	16	16
Gender (male/female; n)	18/14	10/6	9/7
Age (years)	4.3 ± 0.6 (1.0-10.9)	4.7 ± 0.8 (0.6-11.7)	4.2 ± 0.7 (0.5-11.3)
SCORAD	NA	43.13 ± 3.150	36.16 ± 3.645
IgE (kU/L)	NA	639.8 ± 206.0	45.56 ± 14.05
Personal history of atopy (positive/negative; n)	0/32	12/4	3/13

AD, atopic dermatitis; SCORAD, scoring atopic dermatitis; NA, not available. Data are presented as mean ± SEM. Serum IgE levels differed significantly between EAD and IAD (P < 0.0001, Mann-Whitney U-test). No associations between age (Kruskal–Wallis test), SCORAD (Mann-Whitney U-test), and gender (Fisher exact test) were found.

### Flow Cytometry

Peripheral blood mononuclear cells (PBMCs) were isolated from fresh blood by density gradient centrifugation on Ficoll-Paque PLUS (Cytiva). The isolated PBMCs were then analyzed with an 8-color flow cytometer (FACSCanto™II, BD Biosciences, CA, USA) after labeling with the following anti-human fluorochrome-conjugated antibodies: FITC-conjugated CD38 (clone HIT2); APC or FITC-conjugated PD-1 (clone EH12.2H7); FITC-conjugated CD45RA (clone HI100); PE-conjugated CD24 (clone ML5); PE-conjugated ICOS (clone C398.4A); PE-conjugated CCR6 (clone G034E3); PerCP-conjugated CD3 (clone UCHT1); PE/cyanine7-conjugated CD27 (clone O323); PE/cyanine7-conjugated CD4 (clone OKT4); APC-conjugated CD19 (clone HIB19); APC-conjugated CD45RO (clone UCHL1); APC-conjugated CXCR3 (clone G025H7); and Brilliant Violet 421-conjugated CXCR5 (clone J252D4) (all antibodies were from BioLegend, CA, USA). Cell viability was assessed using a live/dead assay (Fixable Viability Dye eFluor 780; eBioscience). All data were collected and analyzed using FlowJo software. The gating strategies of the isotype-matched IgG control for specific cell populations are shown in the supplementary figure (**Figure S1**).

### B Cell Stimulation

PBMCs from HCs and patients with AD were resuspended in culture medium (at 2 × 10^6^ cells/mL) in 48-well flat-bottom plates and stimulated for 48 h at 37 C with CpG (TLR9 ligand, 10 μg/mL; *In vivo*gen) and CD40 ligand (CD40L, 1 μg/mL; BioLegend). Phorbol 12-myristate 13-acetate (PMA, 20 ng/mL; Sigma-Aldrich), ionomycin (1 μg/mL, Sigma-Aldrich), and brefeldin A (BFA, 5 mg/mL, BioLegend) were added for the last 5 h. Next, the cells were harvested and washed twice with phosphate buffer solution (PBS). Single-cell suspensions were then stained for 20 min on ice with predetermined optimal concentrations of APC-conjugated CD19 (BioLegend). Stained cells were washed twice with PBS before fixation and permeabilization in Fixation and Permeabilization Buffer (BD Biosciences). Finally, cells were stained for 30 min with PerCP-conjugated IL-10 (clone JES3-9D7, BioLegend).

### Co-Culture Assays

Human naïve CD45RO^−^CD4^+^ T cells were sorted by Magnetic-Activated Cell Sorting (19555; StemCell Technologies). CD19^+^CD24^hi^CD38^hi^ transitional B cells obtained from HCs or patients with EAD were sorted by flow cytometry (FACSAria™ II, BD Biosciences) and co-cultured (0.25 × 10^5^ cells) for 72 h with naïve CD45RO^−^CD4^+^ T cells (2 × 10^5^ cells) obtained from HC; cells were co-cultured in plates precoated with anti-CD3 (317325; BioLegend) and anti-CD28 (302934; BioLegend) antibodies (1 μg/mL). To polarize Tfh cells (21), recombinant human IL-21 (10 ng/mL, PeproTech), IL-6 (25 ng/mL, PeproTech), 5 μg/mL anti-IFN-γ (506532; BioLegend), anti-IL-4 (500838; BioLegend), and anti-TGF-β (361204; BioLegend) neutralizing antibodies were added to the co-culture system. In some experiments, an anti-IL-10 neutralizing antibody (5 μg/mL, 501427; BioLegend) was added to cultures.

### Measurement of Serum IL-10, IL-21, and Thymus and Activation Regulated Chemokine (TARC) Levels

IL-10, IL-21 and TARC levels in serum samples obtained from patients with AD and from HC were measured using the Luminex assay (RnD, Item No. LXSAHM-15).

### Confocal Microscopy

B cells isolated from human PBMCs by Magnetic-Activated Cell Sorting (17954, StemCell Technologies) were immobilized onto glass slides coated with poly-L-lysine (Sigma-Aldrich). The BCR was labeled with a human IgM+G antibody (Alexa Fluro 546-(Fab)2-anti-Ig(M+G)). This antibody was generated from the F(ab′)2 fragment (Jackson ImmunoResearch, West Grove, PA) using a published protocol (23). Alexa Fluro 546-(Fab)2-anti-Ig(M+G) was incubated with B cells for 30 min on ice before Fc receptor blocking (422301; BioLegend). Then, the B cells were stimulated for 0, 5, 15, or 30 min at 37°C before fixation with 4% paraformaldehyde. In some experiments, B cells were stimulated for 48 h at 37°C with CpG (10 μg/mL) and CD40L (1 μg/mL) to detect activation of phospho-STAT3. The following specific antibodies were used to stain the cells after permeabilization with 0.05% saponin (Sigma): anti-CD38 (ab235118; abcam), anti-CD24 (ab202073; abcam), anti-phospho-STAT3 (9145S; Cell Signaling Technology), anti-phosphotyrosine (05-1050X; Merck Millipore), anti-pBTK (ab52192; abcam), and anti-pCD19 (3571S; Cell Signaling Technology). Images were obtained under a confocal microscope (Nikon A1R) using 405, 488, 546, 647 nm lasers. Colocalization and MFI were determined by the NIS-Elements AR 3.2 software.

### Statistical Analysis

All statistical analyses were performed using GraphPad Prism version 6. The results are expressed as median and interquartile range unless otherwise stated. Spearman’s correlation was used to evaluate the correlations between the two variables. The significance of differences between two groups was evaluated using Mann-Whitney *U*-test, and multiple comparisons were made using Kruskal–Wallis test. A P-value <0.05 was considered statistically significant.

## Results

### The Lower Percentage of IL-10-Producing B Cells in Children With EAD Correlates Inversely With Disease Severity

**Figure 1:**
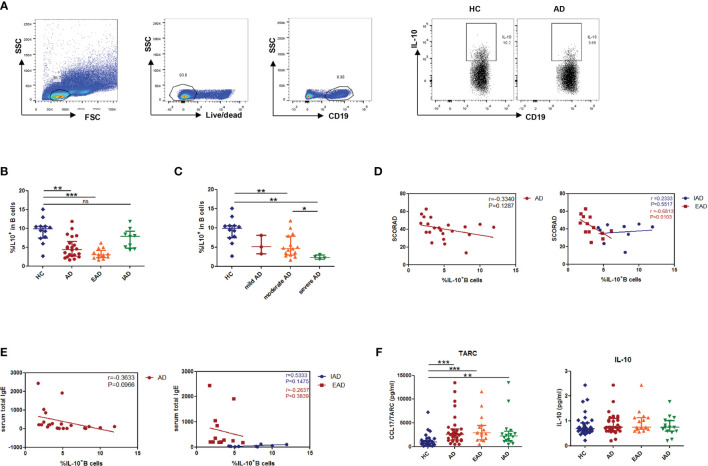
A decreased percentage of IL-10^+^B cells negatively correlates with disease severity in children with EAD. **(A, B)** The percentage of IL-10^+^B cells in patients with EAD (n = 13) is significantly lower than that in healthy controls (n = 12), but not in patients with IAD (n = 9). **(C)** Patients with moderate to severe AD show a lower percentage of Breg cells than those with mild AD. **(D)** The percentage of Breg cells in patients with EAD correlates negatively with SOCRAD. **(E)** There is no significant correlation between the percentage of Breg cells and total serum IgE level in patients with EAD. **(F)** There is no difference between the AD and HC groups with respect to serum levels of IL-10, but there is a significant increase in serum TARC levels in the AD group [HC: n = 32; patients with AD: n = 32, including EAD (n = 16) and IAD (n = 16)]. Data are represented as median and interquartile range. *P < 0.05, **P < 0.01, and ***P < 0.001. ns, not significant (Mann-Whitney *U*-test).

PBMCs from patients with AD or HC were incubated with CpG and CD40L for 48 h, respectively, and then PMA/ionomycin and brefeldin A were added for the last 5 h before the assessment of intracellular IL-10 expression. The results showed that the percentage of CD19^+^IL-10^+^B cells in patients with EAD was significantly lower than that in HC but not in patients with IAD (**Figures 1A, B** and **S2A**). AD was categorized as mild, moderate, or severe in accordance with SCORAD. Patients with moderate to severe AD had a lower percentage of Bregs than those with mild AD (**Figure 1C**), suggesting that Breg cells may be associated with disease severity. However, we found no significant correlation between the percentage of Breg cells and SCORAD in total patients with AD. It is interesting to note that when the classification of IAD and EAD is taken into account, the percentage of Breg cells correlated negatively with SOCRAD (p=0.0103, r=-0.68) in patients with EAD (**Figure 1D**), again suggesting that the relative reduction in percentage of Breg cells in EAD patients shows an inverse correlation with AD severity.

Because most EAD patients have high IgE levels, we examined the correlation between the percentage of Breg cells and total serum IgE levels. There was no significant correlation between these parameters (**Figure 1E**). Furthermore, when we examined serum levels of IL-10 in patients and controls, we found no difference between the AD and HC groups; however, there was a significant increase in serum thymus and activation-regulated chemokine (TARC) levels in the AD group (**Figure 1F**).

### IL-10-Producing B Cells Are Enriched Within the CD19^+^CD24^hi^CD38^hi^ B Cell Subset in Patients With AD

**Figure 2:**
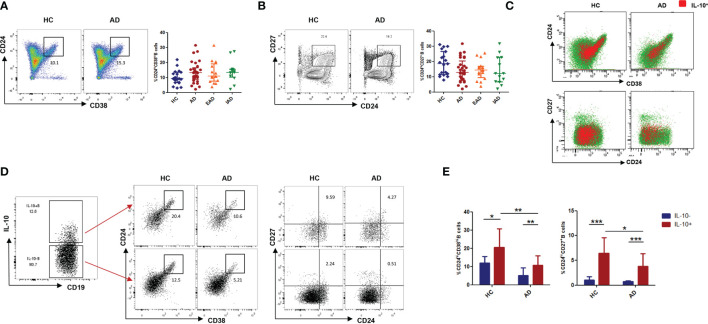
The phenotype of IL-10^+^Breg cells in peripheral blood from patients with AD. **(A, B)** The percentages of CD19^+^CD24^hi^CD38^hi^ transitional B cells and CD19^+^CD24^hi^CD27^+^ memory B cells in patients with AD are normal (HC: n = 19; patients with AD: n = 29). **(C)** Distribution of IL-10^+^B cells within B cell subsets, defined by expression of CD24/CD38 and CD24/CD27, in patients with AD and in healthy controls. **(D, E)** CD24^hi^CD38^hi^ and CD24^hi^CD27^+^ cells were more frequent in IL-10^+^ B cells than in IL-10^-^ B cells (HC: n = 11; patients with AD: n = 13). Data are represented as median and interquartile range. *P < 0.05, **P < 0.01, and ***P < 0.001 (Mann-Whitney *U*-test).

Next, we analyzed the percentages of two phenotypes of Breg cells in peripheral blood and found no significant difference in the percentage of CD19^+^CD24^hi^CD38^hi^ transitional B cells and CD19^+^CD24^hi^CD27^+^ memory B cells between patients and HCs (**Figures 2A, B**). To determine the ability of these two above-mentioned subsets to produce IL-10, we measured expression of CD24/CD38 and CD24/CD27 by IL-10-producing B cells. Expression of CD24/CD38 and CD24/CD27 by IL-10^+^ B cells was higher than that by IL-10^-^ B cells in patients with AD (**Figures 2C–E**); also, the Breg subset was dominated by CD19^+^CD24^hi^CD38^hi^ B cells (**Figure 2E**). Hence, the CD19^+^CD24^hi^CD38^hi^ phenotype was used to denote Breg cells in the current study.

### The Percentage of CXCR5^+^ICOS^+^PD-1^+^ cTFH in Children With EAD Is Higher Than That in IAD and HCs, and Shows an Inverse Correlation With the Percentage of IL-10^+^ Breg Cells

**Figure 3:**
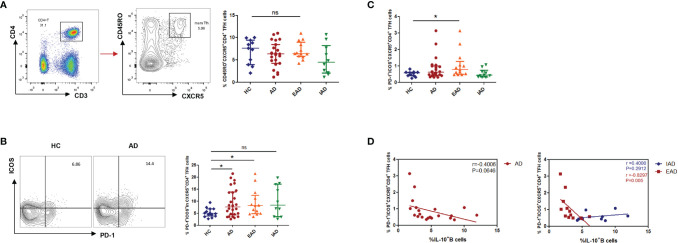
An increased percentage of CXCR5^+^ICOS^+^PD-1^+^ cTFH cells negatively correlates with the percentage of IL-10^+^ Breg cells in children with EAD. **(A)** The percentage of circulating CD3^+^CD4^+^CD45RO^+^CXCR5^+^ Tfh cells is not different between AD patients [n = 23, including EAD (n = 13) and IAD (n = 10)] and healthy controls (n = 11). **(B)** Expression of Tfh-related cell-specific markers by CD4^+^CXCR5^+^ T cells. Percentage of ICOS^+^PD-1^+^ cells within the CD4^+^CXCR5^+^ T cell subset from the two subgroups of AD patients (n = 13 for EAD and n=10 for IAD). **(C)** The percentage of CXCR5^+^ICOS^+^PD-1^+^ Tfh cells is elevated in patients with EAD (n = 13). **(D)** There is a negative correlation between the percentage of CXCR5^+^ICOS^+^PD-1^+^ Tfh cells and the percentage of IL-10^+^ B cells only in EAD patients (n = 13). Data are represented as median and interquartile range. *P < 0.05. ns, not significant (Mann-Whitney *U*-test).

The percentage of CD3^+^CD4^+^CD45RO^+^CXCR5^+^ in the circulating Tfh population was no different in patients with AD than in HC (**Figure 3A**). Tfh cells expressing the costimulatory molecules ICOS and inhibitory receptor PD-1 are considered to be activated. The percentage of ICOS^+^PD-1^+^ cells among CD4^+^CXCR5^+^ Tfh cells was higher in patients with AD than in HC (**Figure 3B**); however, the percentage of CD4^+^CXCR5^+^PD-1^+^ICOS^+^ cTfh cells was higher only in EAD patients (i.e., not in IAD patients) than in HC (**Figure 3C**). In addition, there was a negative correlation (p=0.005, r=-0.8297) between the percentage of CD4^+^CXCR5^+^PD-1^+^ICOS^+^ cTfh cells and IL-10^+^ B cells only in EAD patients (**Figure 3D**). However, there was no correlation between the frequency of circulating Tfh cells and serum levels of IgE or disease severity (SCORAD) (**Figure S3**).

Circulating Tfh cells were classified into three major subsets, each showing differing expression of CXCR3 and CCR6: CXCR3^+^CCR6^−^ type 1 Tfh (Tfh1) cells, CXCR3^−^CCR6^−^ type 2 Tfh (Tfh2) cells, and CXCR3^−^CCR6^+^ type 17 Tfh (Tfh17) cells (24). There was no significant difference between AD patients and HC with respect to the proportion of Tfh1, Tfh2, and Tfh17 cells (**Figure S2B**).

### Breg Cells From Patients With EAD Are Less Able to Suppress Differentiation of Tfh Cells

**Figure 4:**
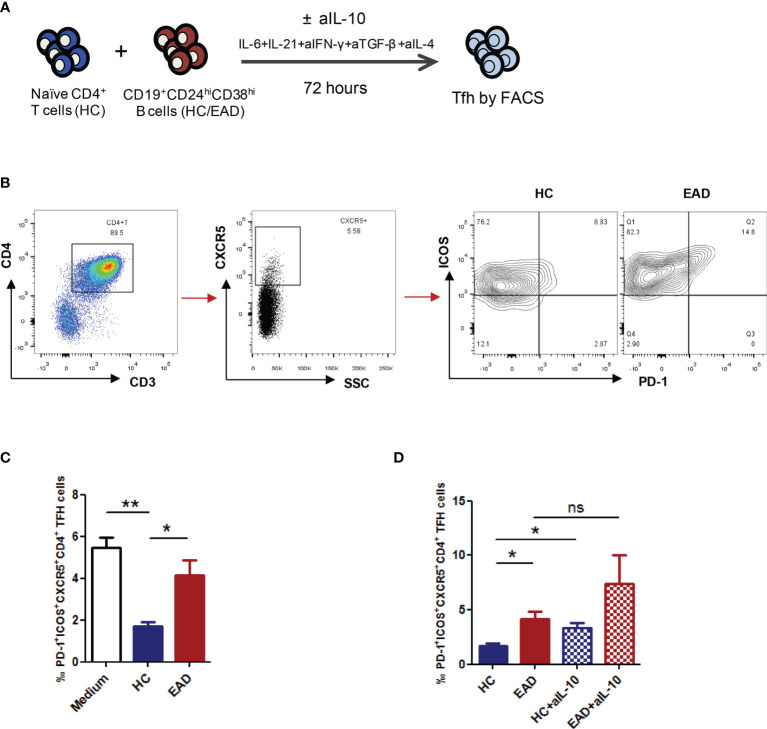
Breg cells from patients with EAD are less able to suppress differentiation of Tfh cells. **(A)** CD19^+^CD24^hi^CD38^hi^ B cells were isolated from HC and patients with EAD by flow cytometry, and CD4^+^ naïve T cells were isolated from HC by MACS. Cells were co-cultured for 72 h under Tfh-polarizing conditions. **(B, C)** The percentage of PD-1^+^ICOS^+^CXCR5^+^CD4^+^ Tfh cells was significantly reduced upon co-culture with Breg cells from HC (n = 4) compared with blank controls (group medium, n = 4), but not upon co-culture with Breg cells from EAD patients (n = 4). **(D)** The suppressive effect of Bregs on Tfh cell differentiation decreased significantly after adding anti-IL-10 neutralizing antibody to the co-culture system (n = 4). Data are expressed as the means ± SEM. *P <0.05 and **P <0.01. ns, not significant (Mann-Whitney *U*-test). Data are representative of three independent experiments.

To assess the ability of Breg cells to suppress Tfh cell differentiation, we analyzed differentiation of CXCR5^+^ICOS^+^PD-1^+^ Tfh cells from CD4^+^ naïve T cells in a co-culture system. We confirmed that CD19^+^CD24^hi^CD38^hi^ B cells were the predominant population of Breg cells in humans (**Figures 2C**). Accordingly, CD19^+^CD24^hi^CD38^hi^ B cells were isolated from HCs and patients with EAD by flow cytometry, and CD4^+^ naïve T cells were isolated by MACS. Cells were co-cultured for 72 h under Tfh-polarizing conditions (21). We found that the percentage of PD-1^+^ICOS^+^CXCR5^+^CD4^+^ Tfh cells was significantly reduced upon co-culture with Breg cells from HC compared with blank controls (CD4^+^ naïve T cells were cultured with medium alone), but not upon co-culture with Breg cells from EAD patients (**Figures 4A–C** and **S4**). This suggests that Breg cells from EAD patients are less able to suppress differentiation of Tfh cells

Concurrently, we added anti-IL-10 neutralizing antibody (aIL-10) to the co-culture system to see whether Breg cells effectively suppress Tfh differentiation in an IL-10-dependent manner. The results showed that the suppressive effect of Bregs on Tfh cell differentiation decreased significantly after adding aIL-10, suggesting that these Breg cells regulate Tfh cell differentiation in an IL-10-dependent manner (**Figure 4D**).

### CD19^+^CD24^hi^CD38^hi^ Breg Cells From Patients With EAD Regulate BCR Signaling Normally But Show Impaired TLR-STAT3 Signaling

**Figure 5:**
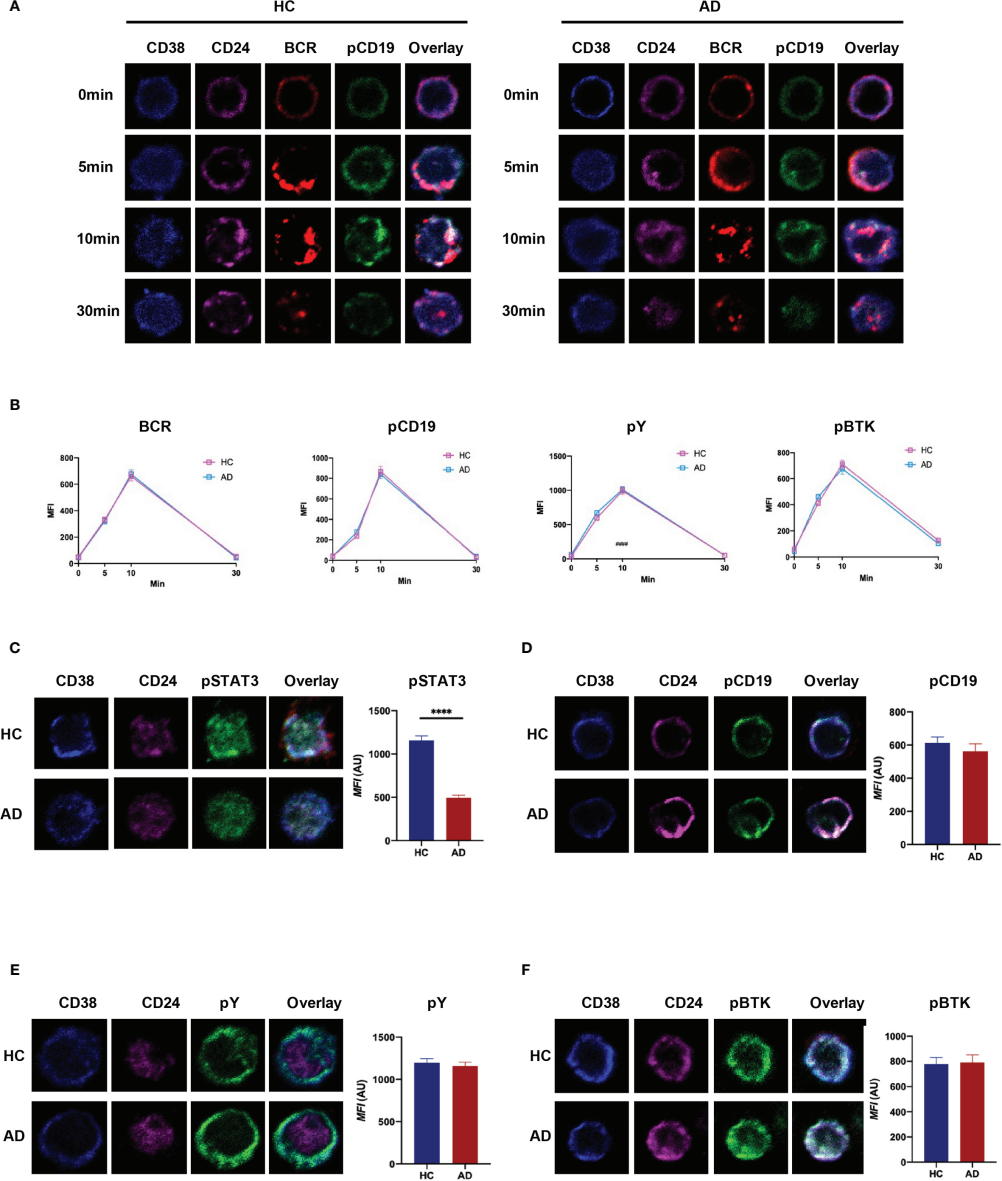
CD19^+^CD24^hi^CD38^hi^ B cells from patients with EAD regulate BCR signaling normally but show impaired TLR-STAT3 signaling. **(A)** B cells were incubated with AF546-(Fab)2-anti-Ig(M+G), to mimic the antigen, for 30 min on ice to label the BCR, and then stimulated at 37°C for different times. **(B)** Analysis of the MFI of BCR, pCD19, pY, and pBtk in CD24^hi^CD38^hi^ B cells after stimulation with soluble antigen. **(C–F)** B cells from patients with EAD or HC were stimulated for 48 h by CpG and incubated with AF546-(Fab)2-anti-Ig(M+G) for 30 min. The MFI of pSTAT3, pCD19, pY and pBtk in CD24^hi^CD38^hi^ B cells was analyzed. Data are expressed as the means ± SEM. ****P < 0.0001 (Mann-Whitney *U*-test). Representative images are shown; in which more than 50 cells were individually analyzed using NIS-Elements AR 3.2 software. Data are representative of three independent experiments.

Current research suggests that naïve B cells differentiate into IL-10-producing Breg precursor cells *via* B-cell receptor (BCR) signaling, while development of mature Breg cells from immature precursor cells is critically dependent on TLR-STAT3 signaling (25, 26). Here, we investigated the ability of Breg cells to respond to BCR and TLR stimulation *in vitro* by monitoring certain key downstream targets (i.e., pY, pCD19, pBTK, and pSTAT3). The results revealed that BCR signaling in CD19^+^CD24^hi^CD38^hi^ B cells from patients with EAD is similar to that in HC (**Figures 5A, B** and **S5**). However, there was a significant reduction in phosphorylation of STAT3 when Bregs from patients with EAD were stimulated for 48 h by CpG (**Figures 5C-F**). This observation indicates that the defective function of Breg cells in patients with EAD may be related to abnormal signal transduction through the TLR-STAT3 pathway, not the BCR pathway.

## Discussion

Breg cells are a population of inhibitory B cells that secrete several suppressive cytokines, including IL-10, TGF-β, and IL-35, which exerts an inhibitory effect on various autoimmune and inflammatory diseases (16, 17, 19). However, the exact role of Breg cells in allergic diseases is still not well understood. Here, we report for the first time that IL-10^+^ Breg cells show abnormal function in children with AD; however, this defect is present only in those with EAD (i.e., not those with IAD). Compared with IAD, EAD is more similar to an allergic disease; patients showed increased levels of serum total IgE or antigen-specific IgE, and an increased peripheral blood eosinophil count; thus, the term of IgE-associated AD was proposed (4). Our study reveals that the percentage of IL-10^+^Breg cells in children with EAD is significantly lower than that in HC, and correlates negatively with disease severity. These findings are consistent with previous literature reports (27). A recent study found no significant correlation between the percentage of IL-10^+^Breg cells and the EASI score of adult patients with AD (28). Combined with the findings above, these data indicate that Breg cells are more involved in the pathogenesis of the allergic subtype of AD rather than that of AD in general. However, we found no significant correlation between the frequency of IL-10^+^ Breg cells and total serum IgE in patients with EAD, although this may be due to larger fluctuations in serum total IgE levels in these patients; also, there was no significant correlation between the amount of serum IgE and disease severity. Some patients with high serum IgE levels (>600 kU/L) were included in this study. There may be multiple lineages of specific IgE in the serum of these patients. In future, it is essential to detect serum antigen-specific IgE and then reassess the correlation between it and the percentage of Breg cells in patients with EAD. Despite the decrease in Breg cell numbers in EAD, serum IL-10 levels in patients with all types of AD were comparable; indicating that serum IL-10 cannot be used as a disease-related clinical biomarker.

Recent studies have emphasized the key function of Tfh cells in promoting and maintaining the germinal center (GC) response during pathogenesis of autoimmune and allergic diseases (11, 29). The GC pathway generates long-lived plasma cells that secrete high affinity IgE. Evidence suggests that Tfh cells, rather than Th2 cells (Th2), play a vital role in controlling IgE production (11, 12). We found that the percentage of CXCR5^+^PD-1^+^ICOS^+^ Tfh cells in patients with EAD, but not IAD, increased and correlated negatively with IL-10^+^ Breg cell numbers. This suggests that a functional defect in IL-10^+^ Bregs may lead to an abnormal Tfh cell population in those with EAD; however, there was no obvious correlation between the percentage of Tfh cells and disease severity or serum total IgE. Tfh cells comprise Tfh1, Tfh2, and Tfh17 cells. Tfh1 cells express T-bet and interferon gamma (IFN-γ); Tfh2 cells express Gata3, IL-4, IL-5, and IL-13; and Tfh17 cells express Rorγt and IL-17A (24). Although studies of total CD4^+^CXCR5^+^ Tfh cells in allergic rhinitis patients show inconsistent results (13, 30), compared with that in healthy subjects the percentage of CXCR3^-^CCR6^-^ Tfh2 cells in AR patients is higher (29). Tfh2 cell numbers correlate positively with the levels of biomarkers of allergic airway inflammation, making it an indicator of progression from AR to AR with asthma (13). However, we found no significant difference in the proportions of Tfh1, Tfh2, and Tfh17 between EAD and HC. Considering that abnormal IL-10^+^ Breg cells may be involved in pathogenesis, and that the percentage of CD19^+^CD24^hi^CD38^hi^ B cells in EAD patients was normal, we speculate that detecting the function of Tfh in AD is more valuable than detecting the phenotype with respect to assessing disease activity. For example, IL4^+^IL-13^+^ Tfh13 and IL-21^+^ Tfh cells are worth studying in patients with AD.

To explore the possible mechanism underlying the defect of IL-10^+^Breg cells in AD, we investigated the two main signaling pathways that regulate IL-10^+^ Bregs development. BCR signaling is the main driver of IL-10^+^ Bregs development because Bregs need to capture antigen to establish an interaction with antigen-specific T cells, which then deliver developmental signals. This was confirmed by the finding that deletion of CD19 led to a significant reduction in IL-10^+^ Bregs (25, 31). Patients with moderate to severe AD show abnormal bacterial colonization or a flora imbalance on the skin surface, which may disrupt the development of Breg cells *via* abnormal antigen-BCR signaling. However, immunofluorescence analysis revealed that BCR signaling in EAD was no different from that in HC. In addition to BCR signaling, activation of TLR-STAT3 signaling is an important step that ensures that B cells are capable of producing IL-10 (26). We found that after culture of B cells cultured with CpG (a stimulator of TLR9) for 48 h, the level of pSTAT3 in Breg cells from patients with EAD decreased when compared with that in Breg cells from HC. Thus, abnormal pSTAT3 signaling induced by TLRs, not abnormal BCR signaling, may contribute to the defect of Breg cells in AD.

In conclusion, our research results demonstrate an important role for IL-10^+^ Breg cells in inhibiting Tfh cell differentiation, suggesting a role in the pathogenesis of AD. These data may facilitate the development of new therapeutic strategies for AD patients.

## Data Availability Statement

The original contributions presented in the study are included in the article/**Supplementary Material**. Further inquiries can be directed to the corresponding authors.

## Ethics Statement

The studies involving human participants were reviewed and approved by Medical Ethics Committee of the Children’s Hospital of Chongqing Medical University. Written informed consent to participate in this study was provided by the participants’ legal guardian/next of kin.

## Author Contributions

JJ and HW designed the study and wrote the manuscript. JJ, SY, XZ, JZ, and XB performed the experiments and analyzed the data. XL, QT, YX, and HW followed up the patients. All authors contributed to the article and approved the submitted version.

## Funding

This work was supported by the National Natural Science Foundation of China (81803140).

## Conflict of Interest

The authors declare that the research was conducted in the absence of any commercial or financial relationships that could be construed as a potential conflict of interest.

## Publisher’s Note

All claims expressed in this article are solely those of the authors and do not necessarily represent those of their affiliated organizations, or those of the publisher, the editors and the reviewers. Any product that may be evaluated in this article, or claim that may be made by its manufacturer, is not guaranteed or endorsed by the publisher.
