# Supplementary material for: Crosstalk Between Circulating Follicular T Helper Cells and Regulatory B Cells in Children With Extrinsic Atopic Dermatitis
Source: Front Immunol. 2021 Nov 30;12:785549. doi: 10.3389/fimmu.2021.785549 (PMC8669722; doi:10.3389/fimmu.2021.785549)
Supplement: Supplementary file 1 [file DataSheet_1.pdf]

Figure S1

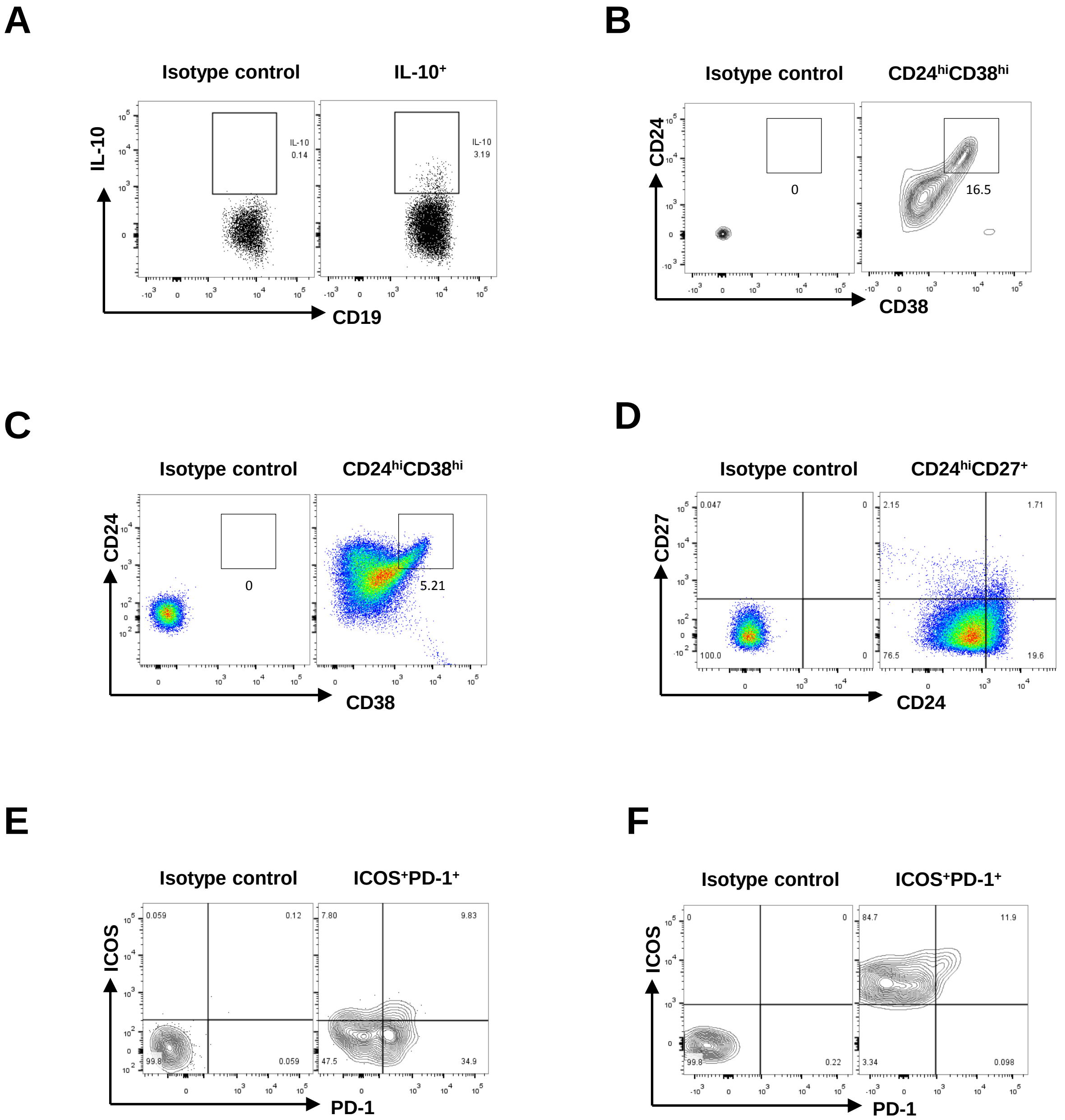

Figure S1. Isotype-matched IgG control for gating in flow cytometry. (A) Isotype-matched IgG control for gating of IL-10$^+$ cells. (B) Isotype-matched IgG control for gating of CD24^{hi}CD38^{hi} cells. (C-D) Isotype-matched IgG control for gating of CD24^{hi}CD38^{hi} cells (C) and CD24^{hi}CD27$^+$ cells (D) in IL-10$^+$ B cells after stimulation. (E) Isotype-matched IgG control for gating of ICOS$^+$PD-1$^+$ cells. (F) Isotype-matched IgG control for gating of ICOS$^+$PD-1$^+$ cells after co-culture for 72 h under Tfh-polarizing conditions.

Figure S2

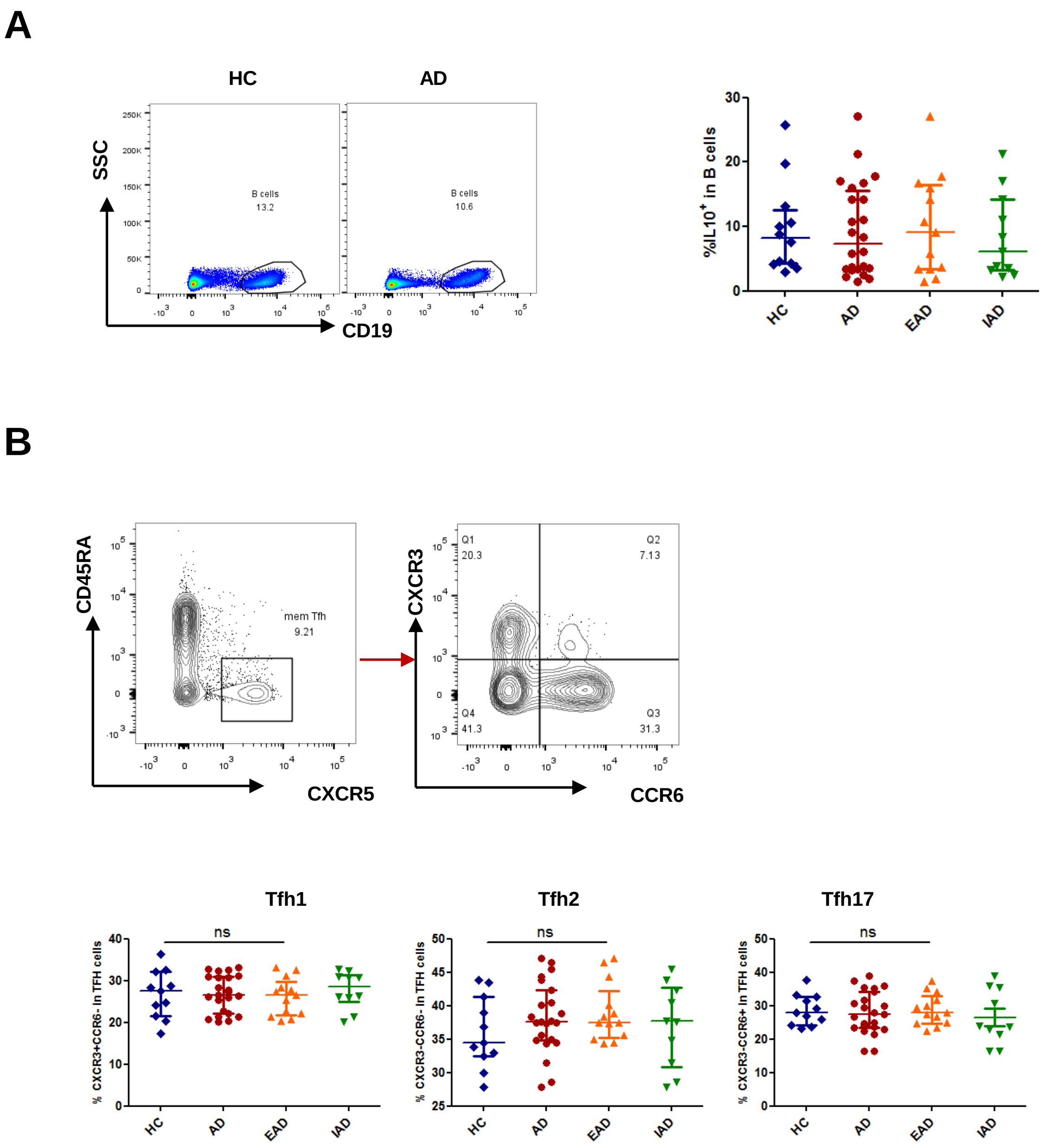

Figure S2. Frequencies of B-cell and Tfh subset in patients with AD. (A)Flow cytometry analysis of $CD19^+$ B cells in patients with EAD and IAD. (B) There is no significant difference in the proportions of Tfh1, Tfh2, and Tfh17 cells between AD patients (n=23) and healthy controls (n=11). Data are represented as median and interquartile range. Comparisons were made using the Mann-Whitney *U*-test.

Figure S3

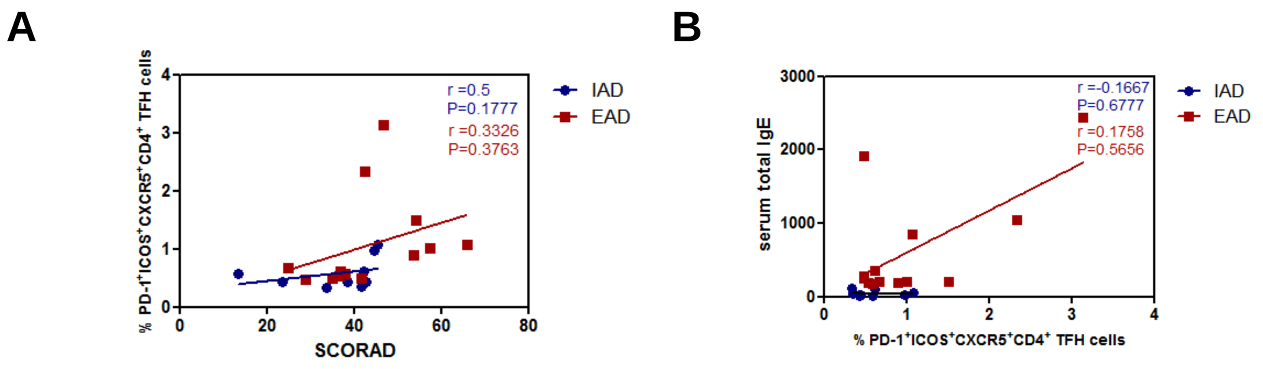

Figure S3. Correlation analysis of Tfh cell frequency with IgE levels and SCORAD. There was no correlation between the frequency of circulating CXCR5$^+$ICOS$^+$PD-1$^+$ Tfh cells and serum levels of IgE (A) or disease severity (SCORAD) (B). Spearman's correlation was used to analyze the correlations between variables.

Figure S4

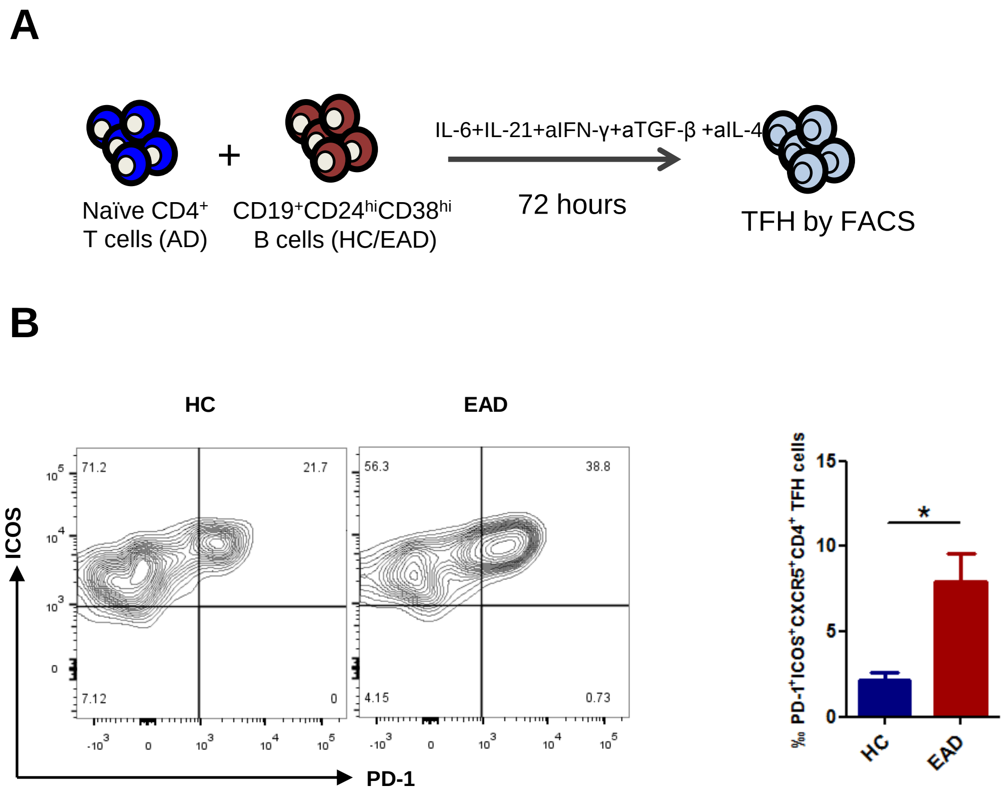

Figure S4. Breg cells from patients with EAD are less efficient in suppressing differentiation of Tfh cells from patients. (A) CD4$^+$ na ïve T cells were isolated from patients with EAD by MACS. **(**B) The percentage of PD-1$^+$ICOS$^+$CXCR5$^+$CD4$^+$ Tfh cells was reduced upon co-culture with Breg cells from HC (n=4) compared with those from EAD patients (n=4). Data are represented as mean ± SEM. *P <0.05 (Mann-Whitney *U*-test).

Figure S5

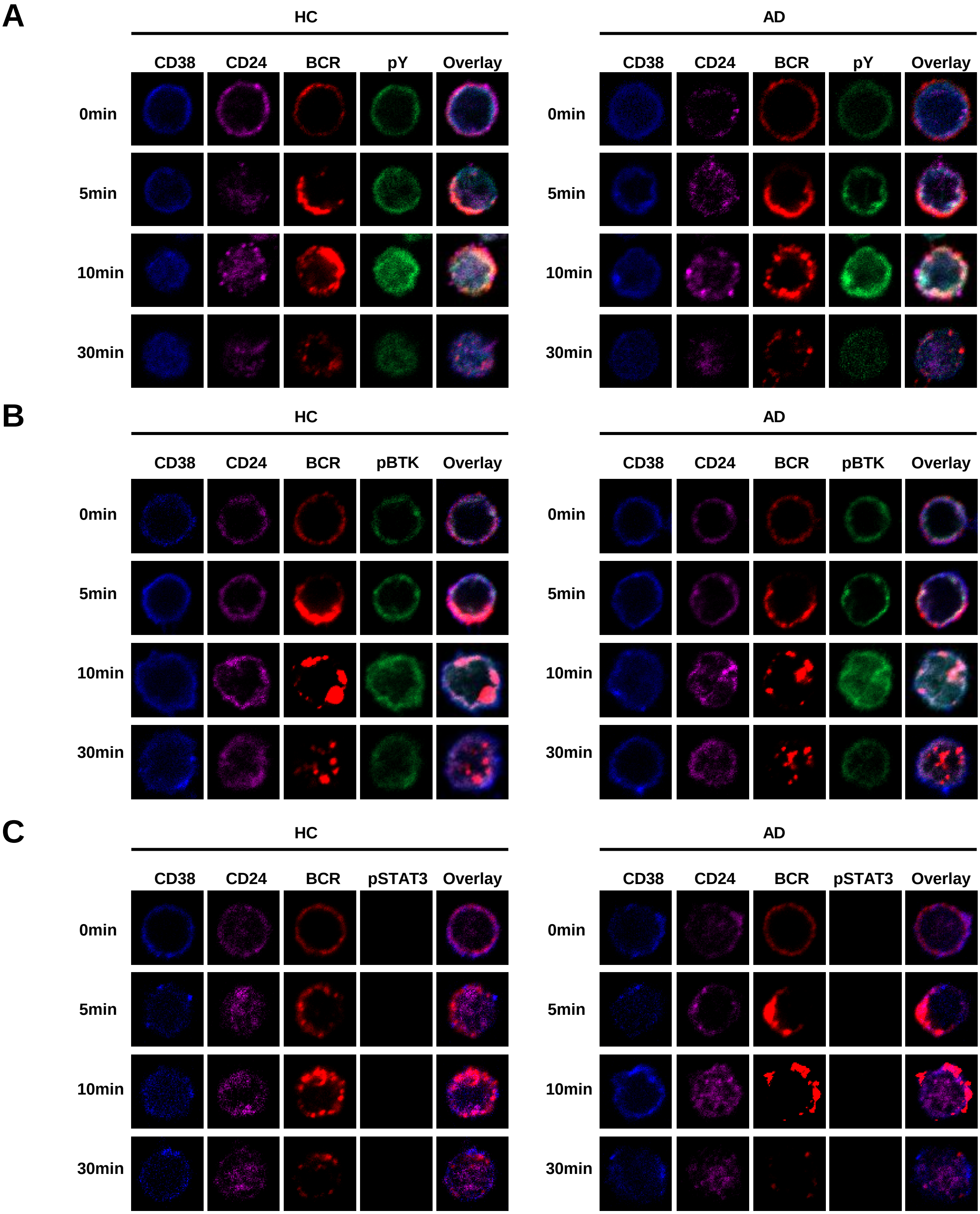

Figure S5. CD19^{+}CD24^{hi}CD38^{hi} B cells from patients with EAD regulate BCR signaling normally. (A-C) B cells were incubated with AF546-(Fab)2-anti-Ig(M+G) for 30 min on ice to label the BCR, and then stimulated at 37℃ for different times. The cells were stained for pY (A), pBtk (B) and pSTAT3 (C), and analyzed using confocal microscopy.
